# Supplementary material for: A proteomic survival predictor for COVID-19 patients in intensive care
Source: PLOS Digit Health. 2022 Jan 18;1(1):e0000007. doi: 10.1371/journal.pdig.0000007 (PMC9931303; doi:10.1371/journal.pdig.0000007)
Supplement: S1 Table — (DOCX) [file pdig.0000007.s002.docx]

|  | **all patients** | | **survived** | | **deceased** | | |
| --- | --- | --- | --- | --- | --- | --- | --- |
| **Number of patients** | 50 | 100 % | 35 | 70 % | 15 | 30 % |  |
| **Sex** |  |  |  |  |  |  |  |
| Female | 15 | 30 % | 12 | 34 % | 3 | 20 % |  |
| Male | 35 | 70 % | 23 | 66 % | 12 | 80 % |  |
| **Age, years** (Median, IQR) | 62 | [54 - 73] | 61 | [54 - 69] | 69 | [56 - 76] |  |
| ≥ 65 | 22 | 44 % | 12 | 34 % | 10 | 67 % |  |
| **BMI** (kg/m2, Median, IQR), n = 49 | 29.4 | [27.4 - 34.7] | 31.0 | [27.4 – 35.1] | 29.0 | [25.7 - 31.2] |  |
| < 25 kg/m2 | 9 | 18 % | 6 | 18 % | 3 | 20 % |  |
| ≥ 25 kg/m2 | 40 | 82 % | 28 | 82 % | 12 | 80 % |  |
| **Pre-existing conditions** |  |  |  |  |  |  |  |
| Charlson’s Comorbidity Index (Median, IQR) | 3 | [1 - 4] | 3 | [1 - 4] | 3 | [3 - 5] |  |
| CCI <3 | 18 | 36 % | 16 | 46 % | 2 | 13 % |  |
| CCI ≥3 | 32 | 64 % | 19 | 54 % | 13 | 87 % |  |
| h/o smoking | 8 | 16 % | 6 | 17 % | 2 | 13 % |  |
| current smoker | 1 | 2 % | 1 | 3 % | 0 | 0 % |  |
| **Outpatient medications** (Median, IQR) | 2 | [1 - 4] | 2 | [1 - 4] | 2 | [1 - 3] |  |
| **Duration of hospital course** (days, median, IQR) | 50 | [33 - 79] | 63 | [44 - 89] | 28 | [16 - 43] |  |
| Proning | 42 | 84 % | 30 | 86 % | 12 | 80 % |  |
| RRT | 36 | 72 % | 23 | 66 % | 13 | 87 % |  |
| ECMO | 19 | 38 % | 12 | 34 % | 7 | 47 % |  |
| ARDS | 49 | 98 % | 34 | 97 % | 15 | 100 % |  |
| Sepsis | 29 | 58 % | 18 | 51 % | 11 | 73 % |  |
| Thromboembolic event | 23 | 46 % | 17 | 49 % | 6 | 40 % |  |
| Cardiopulmonary resuscitation | 5 | 10 % | 4 | 12 % | 1 | 7 % |  |
| **Outcome** |  |  |  |  |  |  |  |
| Deceased (incl. secondary DNR) | 15 | 30 % | - | - | 15 | 100 % |  |
| Secondary DNR | 3 | 6 % | - | - | 3 | 6 % |  |
| Requiring new oxygen therapy after  discharge* | 10 | 29 % | 10 | 29 % | - | - |  |
| Requiring new RRT after discharge* | 5 | 14 % | 5 | 14 % | - | - |  |
| Data are shown in n (%) unless otherwise indicated. IQR - interquartile range, BMI - body mass index, RRT - renal replacement therapy, ECMO - extracorporeal membrane oxygenation, secondary DNR - secondary limitation of therapy in situation of probable unfavorable outcome and according to the presumed patient’s wish    * Deceased patients not included | | | | | | | |

### **S1 Table.** Baseline, treatment, and outcome characteristics of patient cohort with severe COVID-19 receiving maximum therapy at Charité - University hospital Berlin.
