# Supplementary material for: A proteomic survival predictor for COVID-19 patients in intensive care
Source: PLOS Digit Health. 2022 Jan 18;1(1):e0000007. doi: 10.1371/journal.pdig.0000007 (PMC9931303; doi:10.1371/journal.pdig.0000007)
Supplement: S2 Table — Red writing indicates proteins involved in the complement system. Blue writing indicates proteins involved in coagulation. (DOCX) [file pdig.0000007.s003.docx]

| **Gene identifier** | **Protein** | **Relevance score** |
| --- | --- | --- |
| **AHSG** | Alpha-2-HS-glycoprotein, Alpha-2-Z-globulin, Fetuin-A | 1.0 |
| **A2M** | Alpha-2-macroglobulin | 0.84 |
| **PLG** | Plasminogen | 0.67 |
| **F2** | Prothrombin | 0.57 |
| **SERPINA1** | Alpha-1 antitrypsin | 0.57 |
| **SERPINC1** | Antithrombin-III | 0.54 |
| **F12** | Coagulation factor XII | 0.51 |
| **C1QB** | Complement C1q subcomponent subunit B | 0.51 |
| **C1QC** | Complement C1q subcomponent subunit C | 0.47 |
| **F11** | Coagulation factor XI | 0.45 |
| **PF4** | Platelet factor 4 | 0.43 |
| **LPA** | Lysophosphatidic acid receptor 2 | 0.41 |
| **C5** | Complement C5 | 0.35 |
| **TTR** | Transthyretin | 0.32 |
| **VWF** | Von-Willebrand-Factor | 0.31 |
| **F5** | Coagulation factor V | 0.31 |
| **HPX** | Hemopexin | 0.32 |
| **TFRC** | Transferrin receptor protein 1, CD71 | 0.30 |
| **KLKB1** | Plasma kallikrein | 0.30 |
| **C4B** | Complement C4-B | 0.28 |
| **F10** | Coagulation factor X | 0.28 |
| **B2M** | Beta-2-microglobulin | 0.27 |
| **SHBG** | Sex hormone-binding globulin | 0.27 |
| **C1QA** | Complement C1q subcomponent subunit A | 0.27 |
| **SERPING1** | Plasma protease C1 inhibitor | 0.27 |

### **S2 Table.** Top 25 proteins included in the machine learning model, ordered by their estimated ‘relevance’ scores (Methods). Red writing indicates proteins involved in the complement system. Blue writing indicates proteins involved in coagulation.
