# Supplementary material for: A proteomic survival predictor for COVID-19 patients in intensive care
Source: PLOS Digit Health. 2022 Jan 18;1(1):e0000007. doi: 10.1371/journal.pdig.0000007 (PMC9931303; doi:10.1371/journal.pdig.0000007)
Supplement: Attachment — Submitted filename: Response to Reviewers.docx [file pdig.0000007.s006.docx]

# Point by point response

## Reviewer #1:

Demichev et al. report the analysis of plasma proteomes from two cohorts of patients with severe COVID-19. Based on proteome profiles at early timepoints of maximum care the authors derive a predictor of clinical outcome for ICU patients, indicating that proteomics could outperform conventional risk assessment in terms of accuracy. This is a follow-up on a study just published in Cell Systems (ref 13) by the authors using the same data set, focusing on the specific point of predicting an individual’s outcome. As such it is of interest and I can recommend publication upon addressing the comments below.

1) Please indicate the size of the patient cohorts, number of samples and proteomics depth in the abstract as these are important for the interpretation of the statistical analysis.

**Response**: We would like to thank the reviewer for their extensive engagement with our research and appreciate their comments. Following the reviewer’s suggestion we have added these key data to the abstract. The respective section in the abstract now reads:

*Instead, the quantification of 321 plasma protein groups at 349 timepoints in 50 critically ill patients receiving invasive mechanical ventilation revealed 14 proteins that showed trajectories different between survivors and non-survivors.*

2) The introduction states that molecular differences are less pronounced within the same than between different severity groups. Is there any data that could support this?

**Response**: We thank the reviewer for this comment and agree that the sentence as it was phrased in the original version of the manuscript was too vague. Molecular differences in different COVID-19 severity groups have been assessed by numerous studies. In particular the proteomic studies by Shen et al. (Shen et al. 2020) and our own work by Messner et al. and Demichev et al. (Messner et al. 2020; Demichev et al. 2021) found a noticeably homogeneous distribution of molecular markers within the severity groups. This is the basis for the proteomic severity classification, that we have presented in our recent data ressource manuscript by Demichev et al. (Demichev et al. 2021).

According to the suggestion of the reviewer we rephrased the respective paragraph and also added references to support the statement made. The respective section reads now:

*Indeed, testing for the success of a clinical intervention requires classification of divergent clinical trajectories within more homogeneous groups, such as WHO grade 7 patients. At least in COVID-19, this is hampered by the fact that molecular signatures within a group of patients with comparable disease severity are considerably more similar when compared to the differences between mild and severe patients [6,7,15].*

3) Please clarify the relation of the present manuscript to the study just published in Cell Systems. Figure 1 apparently recapitulates results from the Cell Systems study while the authors state that the longitudinal profile is only of limited value for the present study. Therefore, it would appear more appropriate to move panels b and c to Supplementary Material. Also, in the discussion: ‘Here, we show that an increase in specific inflammatory …’ Is this really a result of the present study?

**Response**: We thank the reviewer for this important comment and would like to provide further clarification. In the referenced Data Resource study published in Cell Systems (Demichev et al. 2021) we used proteomes (including their longitudinal profiles) to classify COVID-19 severity, to identify covariations between protein groups and between proteomic and accredited laboratory markers, to analyse the impact of age on the proteome in COVID-19, and to classify therapeutic needs (e.g. invasive mechanical ventilation). However, we did not attempt the possibility of using proteomics to create an outcome predictor.

The present manuscript uses the proteomic data to specifically investigate how longitudinal profiling, as well as the proteomic profile of the patient at the clinical decision point (i.e. first measurement when the patient has been put on the highest applied therapy level) can predict the final outcome, i.e. survival or death, in the most critically ill patients. This prediction was possible due to more complex statistical methods including a machine learning predictor that had not been applied in the previous study. We find that only a subset of the proteins that are differentially concentrated depending on COVID19 severity according to our previous study, are also prognostic and are predicting the outcome from a single time point sample. We have added a clearer explanation to the manuscript, that spells out better the conceptual advance (outcome prediction) this manuscript provides over previous work.

4) It would be helpful to have a summary of the sample cohort akin to Suppl. Table 1 in Figure 1.

**Response**: We thank the reviewer for their suggestion and have adapted Figure 1a in order to include key information about the sample cohort, i.e. cohort size, distribution of sex, distribution of survivors vs non-survivors.

5) Please indicate how many proteins were included in the statistical model. I think it is important to discuss the potential of overfitting given the presumably large number of proteins as compared with the relatively small cohort size.

**Response**: We fully agree with the reviewer on this important issue. In our model we used 57 proteins for survival prediction. The proteins were chosen based on the data quality (at least three peptides detected). Moreover, we used the availability of a targeted mass spectrometric assay (MRM assays) as listed in the MRMAssayDB (Bhowmick, Mohammed, and Borchers 2018) as an indicator that these proteins have been identified as markers in other disease settings previously, which increases the confidence that they are suitable biomarkers.

To ensure that any potential overfitting does not affect benchmark results, we only tested the models on samples (one per patient) which were not used during training. Most importantly, the strongest indicator that our results are not explained by overfitting, is that we achieved an excellent performance of the trained model on a fully independent patient cohort, treated at another hospital in another health care setting.

We have now clarified the above in the manuscript.

We have further demonstrated the predictive power of proteomes also using a different machine learning method (SVM), which we included as Supplementary Figure 1 in the revised version of the manuscript.

6) There is an increasingly large body of serum and plasma proteomics in COVID-19. Could the authors discuss their findings, e.g. the panel of inflammatory and acute phase proteins, more extensively in relation to this work? This would eventually help readers to assess the reproducibility of proteomic profiles.

**Response**: We thank the reviewer for this suggestion and have now included a paragraph that links our results to other proteomic studies in COVID-19 in the discussion section of the revised version of the manuscript. We discuss the identified proteins as markers of disease severity and as predictors of mortality. Moreover we also included a potential overlap with proteins which are elevated in non-COVID-19 sepsis.

The additional paragraphs in the discussion section read:

*Here, we show that an increase in specific inflammatory and acute phase proteins over time (e.g., SAA1;SAA2, CRP, ITIH3, LRG1, SERPINA1, and LBP) is associated with the risk of death from COVID-19, while an increase of kallikrein (KLKB1), kallistatin (SERPINA4), thrombin (F2), apolipoprotein C3 (APOC3), GPLD1, and the protease inhibitor A2M, is associated with survival. Interestingly, we and others have found all of these proteins to also be differentially expressed depending on disease severity in COVID-19 [6,7,10,12,15]. Moreover, there is substantial overlap with a panel of proteins predictive of mortality in COVID-19 identified by Völlmy et al. [42]. Hence, despite only a subset of proteins that are differentially concentrated depending on disease severity predict outcome, this result indicates a high congruence and reproducibility of plasma proteome signatures across studies.*

*SAA1;SAA2, CRP, ITIH3, SERPINA1 are acute phase proteins that are also dysregulated in other inflammatory states including sepsis [43]. Increased LRG1 and LBP as well as decreased A2M [43,44] are indicators of an ongoing immune response, complementing the general pro-inflammatory signature of these predictive proteins. APOC3 and GPLD1 are involved in lipid metabolism, which has been shown to be dysregulated in bacterial pneumonia, thereby associated with unfavourable outcome [45].*

[...]

*A number of proteins identified as outcome predictors have also been shown to be differentially expressed in sepsis, including SAA1, CRP, SERPINA1, KLKB1, and A2M [43], indicating a general inflammatory signature rather than specific markers of COVID-19.*

7) It is customary to provide the underlying mass spectrometry raw data of proteomics studies. I recommend doing the same here or otherwise stating why this cannot be done and any restrictions that apply. Likewise, I would appreciate if the computer code could be shared via GitHub or similar.

**Response**: The situation might not be as simple as it sounds; from the scientific point all data should be public, but the human right of privacy applies; as a human individual, and its health parameters can, in theory, be identified from plasma proteomic data.

However, after carefully reviewing the consent forms and ethics, we believe to be able to provide the proteomic data in a fully anonymised form. We have submitted the data to ProteomeXchange via the PRIDE database with the dataset identifier PXD029009. (**Username:** reviewer_pxd029009@ebi.ac.uk, **Password:** 2gcbdsbo).

## Reviewer #2:

The authors analyzed two small patient cohorts with repeated measurements over time for the prognostic purpose of predicting mortality in severely ill COVID-19 patients. Interestingly, they cite Wynants et al. (2020) but do not seem to follow the related advice in this living review. In particular, bold claims about clinical usefulness require strong evidence and should follow the advice given in the TRIPOD statement.

**Response:** We thank the reviewer for this comment and appreciate their feedback.

First, we would like to comment that the Reviewer states our cohorts are ‘small’. The cohort size used in the study was determined by the scientific question addressed - the survival of critically COVID-19 patients that receive the most intense medical treatment possible, which in this case includes ECMO and additional organ support. It is a common situation that data science would profit from maximally large cohorts. However, in medical practice, other restrictions apply too, and in each medical study, these need to be balanced against each other. In this particular case, we are presenting an ICU study, and any clinical investigation in this space is limited by i) the number of individuals that attract such a severe form of the disease that an ICU treatment is necessary, and ii) the capacity of the health care system can provide for this extreme level of treatment. In that sense, our study is typical for the research field; indeed, better powered than a majority of similar studies conducted.

The ‘exploratory’ cohort was treated at the Charité - Universitätsmedizin Berlin; as one of the largest research hospitals and best resourced hospitals in the world, the inclusion was not limited by the resources available; that means, the cohort size corresponds effectively to the number of individuals that in the given responsibility zone acquired critical COVID-19. In other words, the cohort is about the maximum size that a single centre study cohort can reach. Of course, seen purely through the lens of further increasing statistical power, it would have been nice to have more patients, but our successful validation in an independent cohort shows that the study was sufficiently powered for establishing our predictor.

Second, the independent cohort used for validation originates from a smaller urban area (Innsbruck, Austria) and correspondingly, is smaller, as fewer individuals adopted the severe disease form. However, also the validation cohort was sufficiently sized to test our model.

It is possible that with ‘small’, the Reviewer expresses worries that it is difficult not to overfit ML models when the training set is limited due to the nature of the medical question addressed. Aware of this limitation, we have rigorously tested the performance of the model only on samples (one per patient) that were not used for its training. We moreover tested the model on an independent cohort from another hospital. Thus, the performance of the model, as demonstrated by Figure 2, could not be explained by overfitting, as it achieves an accurate prediction on the independent validation cohort. We have now highlighted this in Methods. In order to add further evidence, we have added an additional analysis, in which we demonstrate the predictive power of proteomes also using a different machine learning method (SVM, Supplementary Figure 1), and obtained a significant performance. The reason for this exercise is not to compare the performance of different ML predictors against each other (for this, the cohort size indeed is too small); but to prove that the proteome contains sufficient biological information, so that disease outcome can be predicted.

We would like to thank the reviewer for suggesting to follow the TRIPOD statement. Together with the revised manuscript we provide a TRIPOD checklist for our manuscript, which shows that we follow all key requirements for transparent reporting of studies developing, validating, or updating a prediction model as proposed by the EQUATOR network.

However, this study suffers from several issues and I do not want to pick on the small number of events as this would be too easy and you would argue that proteomics is costly (in how far is this a re-analysis of already published data?).

**Response:** In our rebuttal, we have addressed all comments and issues the reviewer (and the other reviewers) have brought to our attention.

We would like to state that the costs of proteomic measurements were no limitation for this study; we measured proteomes for all patients in the cohort that reached WHO 7 grade COVID-19 at multiple timepoints.

Also it is not clear to me if information from the future (beyond the cross-sectional time point at maximum treatment level) is included in the model - which would basically lead to a useless model for prediction purposes.

**Response**: We appreciated the comment of the reviewer, but we believe there is no reason for concern. Only the data of the clinical decision point (i.e. the first timepoint / measurement at WHO grade 7 for each particular patient) was included and used for training the predictor, as described in the methods section. We apologize if this was not clear, and have highlighted this in the manuscript.

## Reviewer #3:

To the authors:

Dear Dr. Deminchev, dear Dr. Ralser and all appreciated colleagues,

Thank you for giving me the opportunity to review your interesting paper by Demichev and colleagues entiteled “A proteomic survival predictor for COVID-19 patients in intensive care” following submission to this appreciated journal.

At first, I wanna state that I do not have any conflict of interests and I did not find any concerns with plagiarism and self-plagiarism by the authors. Competing interests of some of the authors are clearly outlined.

Coronavirus disease 2019 (COVID-19) pandemia caused by SARS-CoV2 has affected millions of people worldwide with an unacceptable high mortality rate up to 60 % for critical cases. The course of the disease is highly variable, from asymptomatic course up to rapid deterioration in between hours with fulfilling criteria of severe sepsis. Some factors and parameters have been identified influencing or predicting severity and outcome, but early clinical parameters to delineate and allocate best treatment strategies are still not well established. However, these might be very helpful to distinguish the heterogeneous patient cohort in the emergency unit requiring medical attention and for estimation of associated mortality rate, especially at a time, when resources are limited due to a rise of severely ill patients.

For identification of proteomic parameters (obtained from serum) the authors aimed to identify (independent) biomarkers in two very well characterized longitudinal cohorts of patients with very similar medical treatment at the first time point fulfilling WHO-7 criteria (need for intensive care). Focusing on proteomic signatures is also gainful, since in COVID-19 patients changes on level of metabolites and small molecules are less pronounced due to less affection of cellular metabolic (dys-)function. At the end, a panel of 14 proteins is identified allowing prediction of survival at ICU-admission, which might quite helpful for clinical practice. The signature is similar, but not identical to these observed in other studies by Demichev (another paper of the first author), Geyer, Galbraight and Völlmy, supporting the concept, that proteomic analyses might improve prediction of clinical courses in COVID-19.

Congratulation to these impressive results. All in all, the paper is well written and consistent, nonetheless there are some questions and recommendations, which might improve the quality of the paper and should be handled prior to final decision of acceptance:

- A short figure in supplementary data section about distribution (timely and individually) of the 321 proteins might increase clarity of the data.

**Response**: We would like to thank the reviewer for the in-depth look in our work and useful suggestions. The dataset used to create the predictor has been published separately, as a resource to understand COVID-19 disease progression (Demichev et al. 2021). We would kindly direct the reader to the respective parts of the previously published work (Methods and Supp. Fig. 1 and 2) for detailed information about patients, individual sampling time points, and protein abundances. We have now clearly referenced these supplementary figures from (Demichev et al. 2021) in the main text of the manuscript.

- There is only a small body of information with respect of dealing with outliers.

**Response**: We thank the reviewer for this comment. We have now indicated in Methods that we specifically chose to rely on nonparametric statistical methods in order to reduce the influence of outliers on the analysis of results.

- In the discussion section, a paragraph about structural and functional relationship (similarities and differences) would be helpful.

**Response**: According to the suggestion of the reviewer we have added a paragraph with additional information on the functional relationship between the observed proteins and other inflammatory states such as sepsis in the discussion. This paragraph reads:

*SAA1;SAA2, CRP, ITIH3, SERPINA1 are acute phase proteins that are also dysregulated in other inflammatory states including sepsis [43]. Increased LRG1 and LBP as well as decreased A2M [43,44] are indicators of an ongoing immune response, complementing the general pro-inflammatory signature of these predictive proteins. APOC3 and GPLD1 are involved in lipid metabolism, which has been shown to be dysregulated in bacterial pneumonia, thereby associated with unfavourable outcome [45].*

- Also in discussion section a paragraph on overall quality of identified biomarkers would be helpful – are these (un)specific danger signals during host response towards SARS-CoV2 or also other infectious agents as well as might they function as unspecific mortality markers?

**Response**: In addition to the above mentioned paragraph we have included a section that cross-references differential expression of proteins with other diseases including sepsis. The overlap of some of the markers with proteins differentially expressed in sepsis indicates a more general inflammatory signature.

*A number of proteins identified as outcome predictors have also been shown to be differentially expressed in sepsis, including SAA1, CRP, SERPINA1, KLKB1, and A2M [43], indicating a general inflammatory signature rather than specific markers of COVID-19.*

## References

Bhowmick, Pallab, Yassene Mohammed, and Christoph H. Borchers. 2018. “MRMAssayDB: An Integrated Resource for Validated Targeted Proteomics Assays.” *Bioinformatics*  34 (20): 3566–71.

Demichev, Vadim, Pinkus Tober-Lau, Oliver Lemke, Tatiana Nazarenko, Charlotte Thibeault, Harry Whitwell, Annika Röhl, et al. 2021. “A Time-Resolved Proteomic and Prognostic Map of COVID-19.” *Cell Systems* 12 (8): 780–94.e7.

Messner, Christoph B., Vadim Demichev, Daniel Wendisch, Laura Michalick, Matthew White, Anja Freiwald, Kathrin Textoris-Taube, et al. 2020. “Ultra-High-Throughput Clinical Proteomics Reveals Classifiers of COVID-19 Infection.” *Cell Systems* 11 (1): 11–24.e4.

Shen, Bo, Xiao Yi, Yaoting Sun, Xiaojie Bi, Juping Du, Chao Zhang, Sheng Quan, et al. 2020. “Proteomic and Metabolomic Characterization of COVID-19 Patient Sera.” *Cell* 182 (1): 59–72.e15.
