# Supplementary material for: A proteomic survival predictor for COVID-19 patients in intensive care
Source: PLOS Digit Health. 2022 Jan 18;1(1):e0000007. doi: 10.1371/journal.pdig.0000007 (PMC9931303; doi:10.1371/journal.pdig.0000007)
Supplement: Attachment — Submitted filename: Response to reviewer.docx [file pdig.0000007.s007.docx]

PDIG-D-21-00015R1
A proteomic survival predictor for COVID-19 patients in intensive care
PLOS Digital Health

Dear Dr. Frasch,

Thank you for inviting us to submit a second revision of our article for your kind consideration.

Please find our rebuttal letter as response to the comment of Reviewer #2 below, as well as a marked-up copy or our manuscript and a clean version attached.

We also changed the file type of the supplementary figure to ‘Supporting information’ as requested.

We look forward to hearing back from you.

Sincerely,

Florian Kurth
on behalf of all Co-authors.

6. Review Comments to the Author

**Reviewer #2**: Thank you for your comments. The authors do not seem to realize that the limiting sample size factor are the number of indiduals who died (i.e. 15 and 5!). Moreover, if only information from the cross-sectional time point is used for the prediction why is there all this additional processing involved (e.g. why parenclitic networks, did you or did you not impute values - again this is not clear to me but it is highly relevant when putting such an algorithm into practice). As a compromise I suggest to please tone down the text in the places where clinical applicability of the results is implied. The study as itself is interesting in particluar when it comes to biology.

**Response:** We thank the reviewer for having studied our revision. We agree that a higher number of negative outcomes in the exploratory cohort would have strengthened the confidence in the predictor; but at the same time, the argument remains that ICU cohorts are limited in size without us being able to change the situation. Having said that, our results validated in an independent cohort, which gives confidence in our results despite the ICU-typical small sample size.

To address the point of the reviewer, we have changed the manuscript as suggested, and are toning down our claims. We now explicitly state that a further validation in prospective cohorts is necessary before clinical utility is robustly validated.

The time point we use for the prediction is the one where the predictor would be most useful to apply, and that is the first time point at the highest treatment level. We have established in previous work (Demichev et al, Cell Systems 2021) that the early inflammatory response causes the strongest proteomic signature in the disease progression. We have added this information to the text.

We note that during the first revision we indicated in Methods that imputation was indeed used to prepare data for survival prediction (minimal value imputation), both via parenclitic networks and SVM. This is one of the standard practices when processing proteomics data. Standardisation (scaling the data to obtain zero mean and unit variance for each variable) is likewise a standard practice in machine learning. No additional processing was applied.

Changes:

We changed the wording of the manuscript on several occasions, e.g.

*The results are currently based on a comparatively small number of patients with adverse outcome. Given the naturally small sample sizes of ICU cohorts and the exploratory character of our study, findings will have to be validated in larger cohorts, before further steps can be undertaken to translate our findings into clinical practice in the future.*

And

*The findings warrant further prospective assessment of proteomic predictors and the described models in larger cohorts.*

in the discussion section. Please refer to the marked-version for all changes made.
